# Effects of Nogo-A and its receptor on the repair of sciatic nerve injury in rats

**DOI:** 10.1590/1414-431X2020e10842

**Published:** 2021-05-31

**Authors:** Junjie Jiang, Yuanchen Yu, Zhiwu Zhang, Yuan Ji, Hong Guo, Xiaohua Wang, Shengjun Yu

**Affiliations:** 1 Department of Hand Surgery, Yantaishan Hospital, Yantai, China; 2 Department of Clinical Laboratory, Yantaishan Hospital, Yantai, China; 3 Yantai City Municipal Government Hospital, Yantai, China

**Keywords:** Neurite outgrowth inhibitor-A, NgR, Sciatic nerve transection, Spinal cord

## Abstract

Regeneration of injured peripheral nerves is an extremely complex process. Nogo-A (neurite outgrowth inhibitor-A) inhibits axonal regeneration by interacting with Nogo receptor in the myelin sheath of the central nervous system (CNS). The aim of this study was to investigate the effects of Nogo-A and its receptor on the repair of sciatic nerve injury in rats. Sprague-Dawley rats (n=96) were randomly divided into 4 groups: control group (control), sciatic nerve transection group (model), immediate repair group (immediate repair), and delayed repair group (delayed repair). The rats were euthanized 1 week and 6 weeks after operation. The injured end tissues of the spinal cord and sciatic nerve were obtained. The protein expressions of Nogo-A and Nogo-66 receptor (NgR) were detected by immunohistochemistry. The protein expressions of Nogo-A, NgR, and Ras homolog family member A (RhoA) were detected by western blot. At 1 week after operation, the pathological changes in the immediate repaired group were less, and the protein expressions of Nogo-A, NgR, and RhoA in the spinal cord and sciatic nerve tissues were decreased (P<0.05) compared with the model group. After 6 weeks, the pathological changes in the immediate repair group and the delayed repair group were alleviated and the protein expressions decreased (P<0.05). The situation of the immediate repair group was better than that of the delayed repair group. Our data suggest that the expression of Nogo-A and its receptor increased after sciatic nerve injury, indicating that Nogo-A and its receptor play an inhibitory role in the repair process of sciatic nerve injury in rats.

## Introduction

In recent years, although central sensitization has been reported under recognized neuropathic conditions, there is a lack of information on the acute brain activation pattern of peripheral nerve injury (1). Neuropathic pain is still a major global health problem. Peripheral nerve injury is a common clinical disease that causes the partial loss of segmental movement and sensory and autonomic nervous function, placing a heavy burden on patients and their families (2). Therefore, increased attention has been paid to the treatment of sciatic nerve injury. The injury of sciatic nerve is related to the change of gene expression level (3). However, the role of these up-regulated or down-regulated genes remains unclear. Understanding the molecular mechanism of the occurrence and development of sciatic nerve injury is a prerequisite for the development of effective treatment for this highly prevalent disease.

Currently, the repair and regeneration technology of injured peripheral nerve has been significantly improved (4,5). Nogo-A (neurite outgrowth inhibitor-A) is a member of the reticulin protein family and exists in neurons and oligodendrocytes (6). Nogo-A inhibits axonal regeneration by interacting with Nogo receptor (NgR) in the myelin sheath of the central nervous system (CNS) (7,8). However, the function of Nogo-A in neurons remains unclear. As the main receptor of Nogo-A, NgR mediates its effects on Ras homolog family member A (RhoA) and downstream pathways, resulting in collapse of growth cone and inhibition of oligodendrocyte differentiation (9–11).

Previous studies have shown that neutralizing Nogo-A inhibition can improve the functional recovery after spinal cord injury (12). Nogo-A activation of RhoA has also been shown to be important during cerebral infarction (13). The whole effect of hyperbaric oxygen can reduce the expression of Nogo-A and its pathway, and help to inhibit the immediate regeneration after global cerebral ischemia (14,15). However, there is no information about the effect of Nogo-A, NgR, and RhoA on sciatic nerve recovery after injury.

Therefore, the aim of this study was to investigate the effects of Nogo-A and its receptor on the repair of sciatic nerve injury in rats.

## Materials and Methods

### Animals

Eight-week-old male Sprague-Dawley rats (180±20 g) were purchased from Jinan Pengyue Experimental Animal Co., Ltd. (SCXK (Lu) 20190003, China). All rats were maintained at the standard conditions of temperature range (23±2°C), average humidity (55±5%), light-and-dark cycle of 12 h, and eating and drinking *ad libitum*. Animal experiments followed the NIH guidelines (NIH pub. No. 85-23, revised 1996) and have been approved by the Yantaishan hospital animal protection and use committee (2018-14).

### Establishment of sciatic nerve injury model in rats

The rats were anesthetized by intraperitoneal injection of 3% pentobarbital sodium (50 mg/kg). The skin was disinfected with 2% iodine tincture, and deiodinated by 75% alcohol. The incision from the left hip to the back of the thigh was about 5 cm long. After skin incision, the sciatic nerve was separated and exposed at the anterior muscle space of biceps femoris. If necessary, some superficial gluteal muscles were cut off to expose the proximal end of the sciatic nerve to the piriformis outlet and the distal end to the middle and lower 1/3 of the thigh. Sciatic nerve was cut off at 0.8 cm from piriformis muscle outlet. A sharp blade was used to cut the sciatic nerve once to avoid saw contusion. After operation, the wound was washed with sterile normal saline and the skin was sutured.

### Animal groups

Ninety-six rats were randomly divided into 4 groups (n=24): 1) control group: only the sciatic nerve was exposed without injury; 2) sciatic nerve transection group (model): 1 cm of sciatic nerve was removed at 0.8 cm from piriformis muscle outlet to simulate long nerve defect; 3) immediate repair group: the sciatic nerve was cut off at 0.8 cm from the piriformis outlet, and then the epineurium was sutured with 10/0 non-invasive suture under the operating microscope to repair the severed sciatic nerve; and 4) delayed repair group: the sciatic nerve was cut off 0.8 cm at the exit of piriformis muscle, and the distance between the two cut ends was more than 1 cm. After 32 days, the epineurium was sutured with 10/0 non-invasive suture under the operating microscope to repair the severed sciatic nerve.

### Samples collection

At 1 week and 6 weeks after injury (or operation), 12 rats in each group were selected. Six rats were euthanized using 1% pentobarbital sodium (150 mg/kg). The injured end tissues of the spinal cord and sciatic nerve of the corresponding segments were placed in an Eppendorf tube and frozen in liquid nitrogen for western blot detection. The other 6 rats were perfused with normal saline and 4% paraformaldehyde through the left ventricle to the ascending aorta, and tissues of injured spinal cord and sciatic nerve were obtained, fixed with 4% paraformaldehyde for 24 h, and paraffin embedded for histopathological and immunohistochemical detection.

### H&E staining

The thickness of paraffin sections was 5 μM. The slices were dewaxed with xylene and hydrated with ethanol. Sections were dyed with hematoxylin (Solarbio, China) for 5 min and washed with tap water. After 30 s of ethanol differentiation with hydrochloric acid, the sections were soaked in tap water for 15 min, and then placed in eosin dye (Solarbio) for 2 min. The tissues were observed under an Olympus optical microscope (BX51, Olympus, Japan) and histomorphological analysis was performed.

### Tissue immunohistochemistry staining

Tissues were embedded in paraffin and sectioned, and then dewaxed with xylene and hydrated with gradient ethanol solution. H_2_O_2_ methanol solution (3%) was added for inactivation treatment for 20 min, high temperature antigen in citrate buffer solution (pH 6.0) was used for thermal repair for 10 min, and 5% BSA was used for blocking treatment for 20 min. Rabbit anti rat Nogo-A (1:200, orb337377, Biorbyt, UK) and NgR (1:500, orb185998, Biorbyt) polyclonal antibodies were added and reacted overnight at 4°C. After rewarming, goat anti-rabbit IgG labeled with horseradish peroxidase (1:1000, orb21465, Biorbyt, UK) was incubated with the secondary antibody. After DAB staining, slides were dyed again, then dehydrated, cleared, and finally sealed. The slides were observed under the optical microscope (400×, Olympus). Analysis was done with ImageJ software (NIH, USA), and the results are reported as the percentage of positive cells (%).

### Western blotting

Tissue samples were dispersed mechanically and centrifuged (12,000 *g*, 10 min, 4°C). Then, the supernatant was collected, and the concentration of the supernatant was detected by a BCA kit (Solarbio). The 40-μg protein sample was mixed with 10% SDS gel loading buffer (1:1), and heated at 95°C for 5 min to denature the protein. The samples were transferred to PVDF (Merck, Germany) for 30 min at 80 V, and sealed with tris-buffered saline plus Tween 20 (TBST) solution containing 5% skimmed milk powder for 1 h at 4°C. Nogo-A, NgR, and RhoA were diluted with TBST solution containing 3% bovine serum protein, and polyclonal antibody of β-actin was reacted overnight at 4°C. All antibodies used are listed in Table 1. After rewarming, goat anti-rabbit IgG labeled with horseradish peroxidase (HRP) was incubated for 1 h at room temperature, washed, and stained with ECL luminescent substrate for 5 min. The protein expression level was standardized by β-actin, and scanned and quantified by ImageJ software (USA).

**Table 1:** Antibodies used in this study.

Antibody	Source	Dilutions	Company
β-actin	Rabbit	1:2000	orb178392, Biorbyt, UK
Nogo-A	Rabbit	1:1000	orb337377, Biorbyt, UK
NgR	Rabbit	1:500	orb285998, Biorbyt, UK
RhoA	Rabbit	1:500	orb228210, Biorbyt, UK
IgG	Rabbit	1:1000	orb21465, Biorbyt, UK

### Data analysis

SPSS 19.0 software (IBM, USA) was used for data processing. The data are reported as means±SD. One-way analysis of variance (ANOVA) was used to compare the data between groups, and LSD test was used for *post hoc* analysis. All data were analyzed using Shapiro-Wilk normality test. P<0.05 was considered to be statistically significant.

## Results

### H&E staining

**Figure 1:**
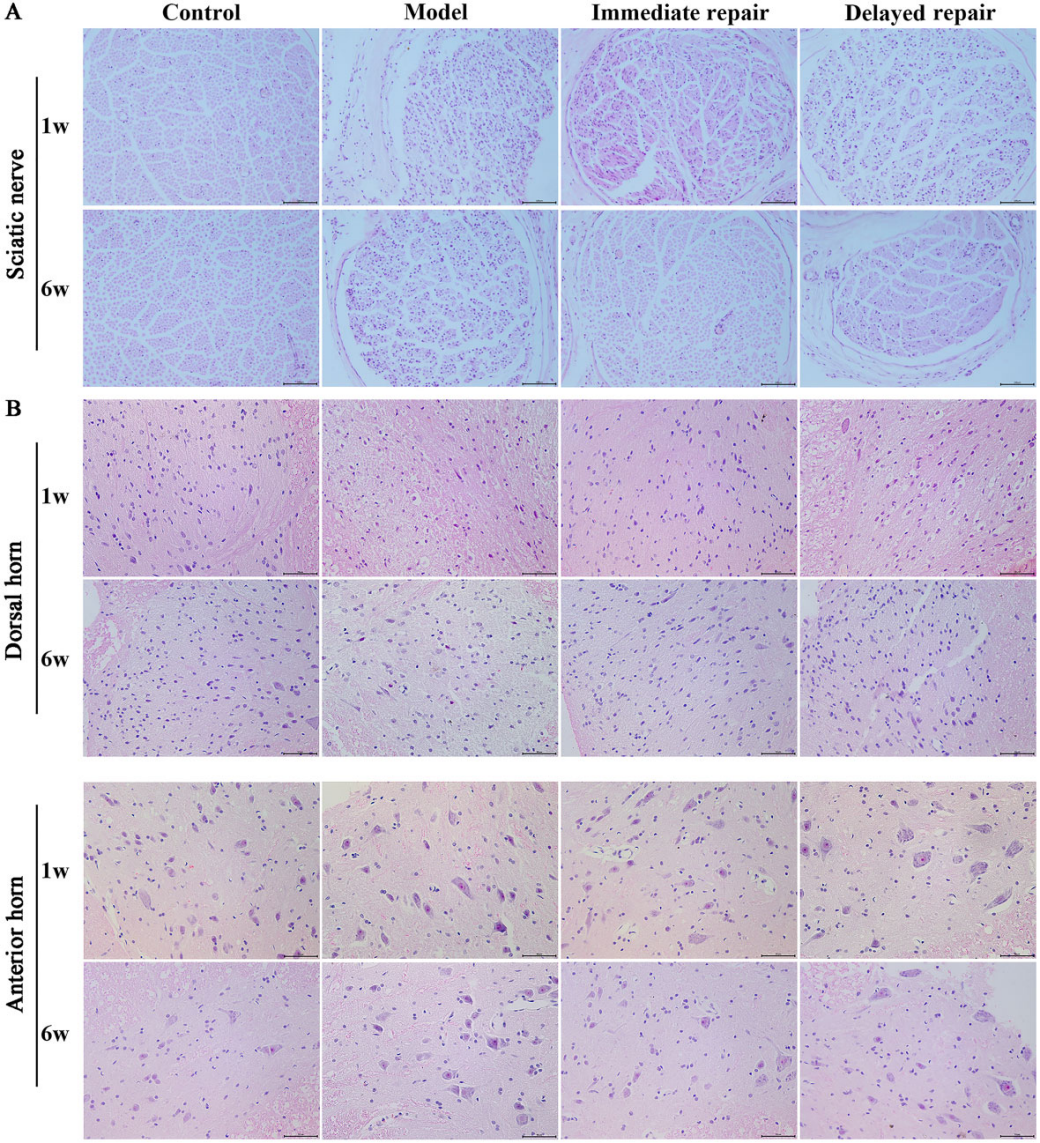
H&E staining was used to observe the pathological changes of the sciatic nerve (**A**) and spinal cord (**B**) after sciatic nerve injury in rats of different groups. Panel A, magnification 200×; scale bar 100 μm; panel B, magnification 400×; scale bar 50 μm.

As shown in Figure 1A, there was no inflammatory cell infiltration at each time point in the control group, and the nerve fibers were arranged orderly, the tissue was dense, and the axon and myelin sheath had good continuity. One week after operation, the nerve fibers in the sciatic nerve transection group were disorderly arranged, some axons disappeared, vacuolar degeneration appeared, most of myelin sheath disintegrated, and edema and inflammatory cell infiltration could be seen. In the immediate sciatic nerve repair group, tissue fibers proliferated and the tissues showed signs of recovery. At 6 weeks after operation, the condition of all operation groups was improved. The pathological improvement of the immediate repair group was better than the delayed repair group. As shown in Figure 1B, one week after operation, there were more normal neurons in the posterior and anterior horn of the spinal cord in the sham operation group, with normal structure. The axons, dendrites, and nuclei of neurons were clear, and the nucleoli were located in the center. In the model group and delayed repair group, there was extensive vacuolation, necrosis, and pyknosis of neurons in the posterior and anterior horn of the spinal cord. The histological changes of spinal cord in the immediate repair group were less than those in the model group. At 6 weeks after operation, the number of normal neurons in the posterior and anterior horn of the spinal cord increased in the model group and the delayed repair group, and neuronal necrosis and nuclear pyknosis were improved. In the immediate repair group, the motoneurons were preserved to a greater extent, and the degree of spinal cord injury was less.

### Protein expressions of Nogo-A and NgR in sciatic nerve

**Figure 2:**
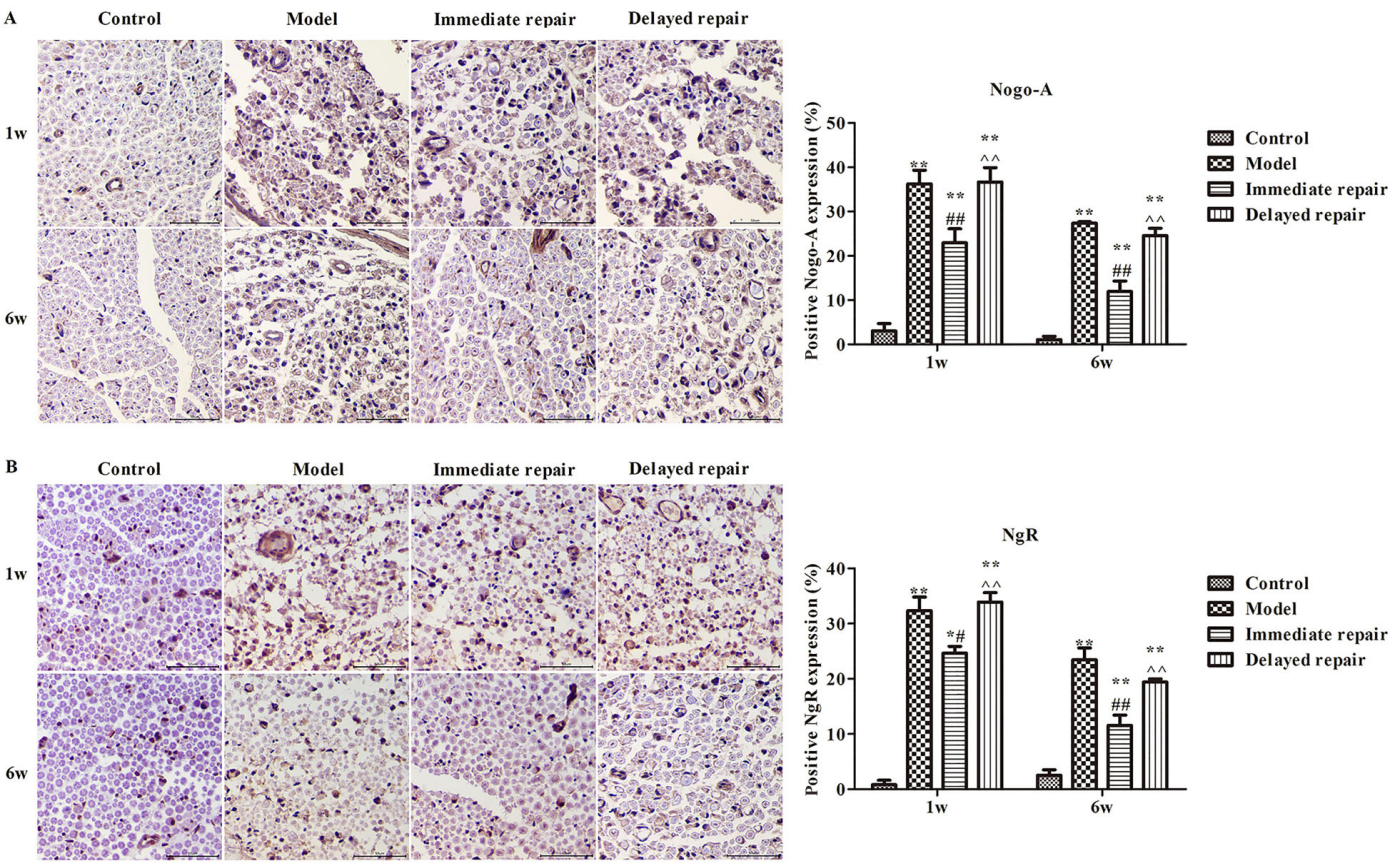
The expressions of Nogo-A (**A**) and NgR (**B**) were detected in sciatic nerve after sciatic nerve injury in rats of different groups by immunohistochemistry. Magnification 400×, scale bar 50 μm. The data are reported as means±SD. *P<0.05, **P<0.01 compared with control group; ^#^P<0.05, ^##^P<0.01 compared with model group; ^^P<0.01 compared with immediate repair group (ANOVA followed by LSD test).

Compared with the control group, the protein expressions of Nogo-A (Figure 2A) and NgR (Figure 2B) in sciatic nerve in the other three groups were significantly increased after 1 week and 6 weeks from operation. Compared with the model group, the protein expressions of Nogo-A and NgR in the immediate repair group were significantly decreased (P<0.05).

### Protein expressions of Nogo-A and NgR in spinal cord

**Figure 3:**
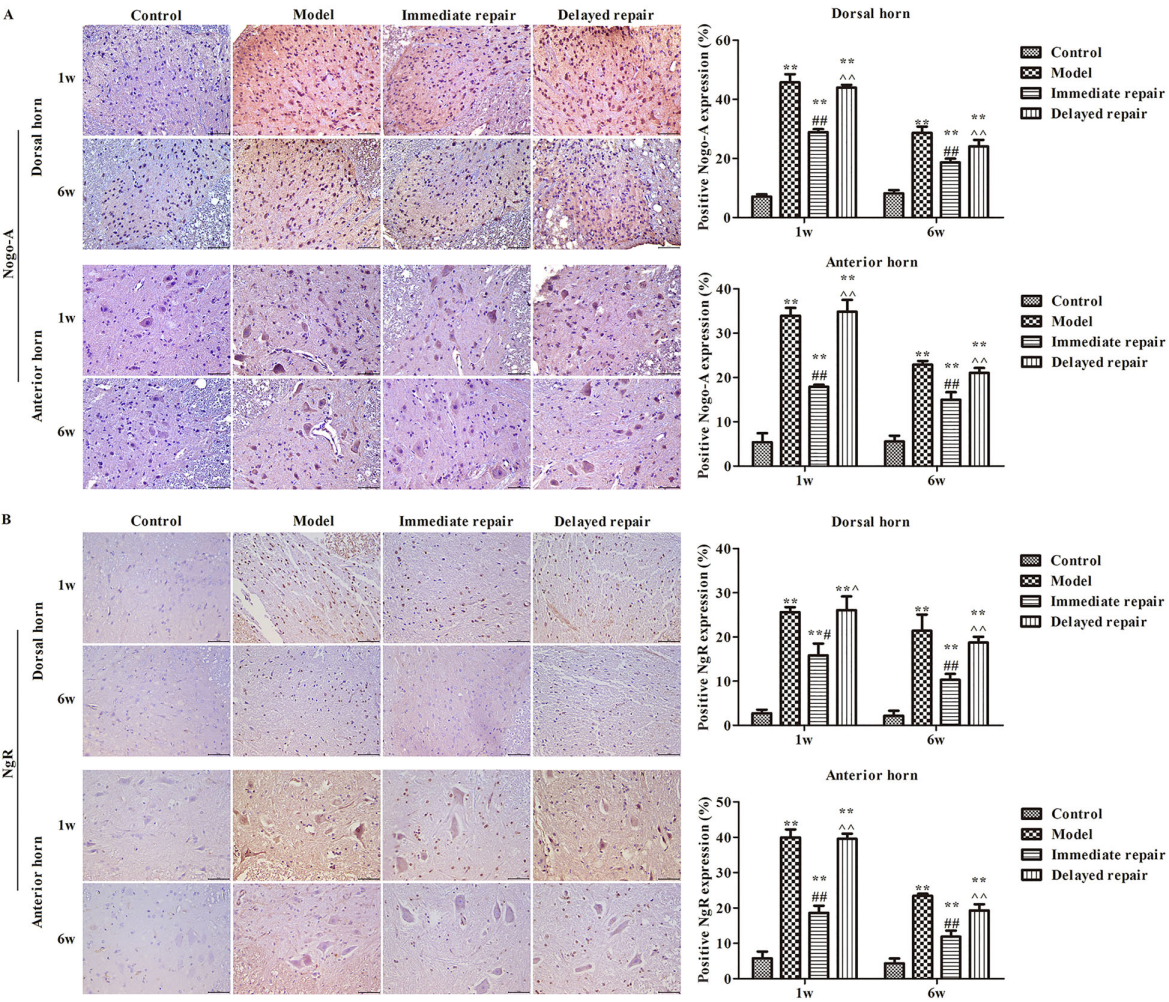
The expression of Nogo-A (**A**) and NgR (**B**) in the anterior horn and posterior horn of the spinal cord after sciatic nerve injury in rats of different groups was detected by immunohistochemistry. Magnification 400×, scale bar 50 μm. The data are reported as means±SD. **P<0.01, compared with control group; ^#^P<0.05, ^##^P<0.01 compared with model group; ^P<0.05, ^^P<0.01 compared to immediate repair group (ANOVA followed by LSD test).

As shown in Figure 3, at 1 week and 6 weeks after operation, the protein expressions of Nogo-A (Figure 3A) and NgR (Figure 3B) in the spinal cord were significantly increased in the other three groups compared with the control group both in dorsal horn and anterior horn (P<0.05). Similar to the sciatic nerve, the expressions of Nogo-A and NgR in the immediate repair group were significantly decreased compared with the model group, and the immediate repair group was lower than that in the delayed repair group (P<0.05).

### Protein expressions of Nogo-A, NgR, and RhoA

**Figure 4:**
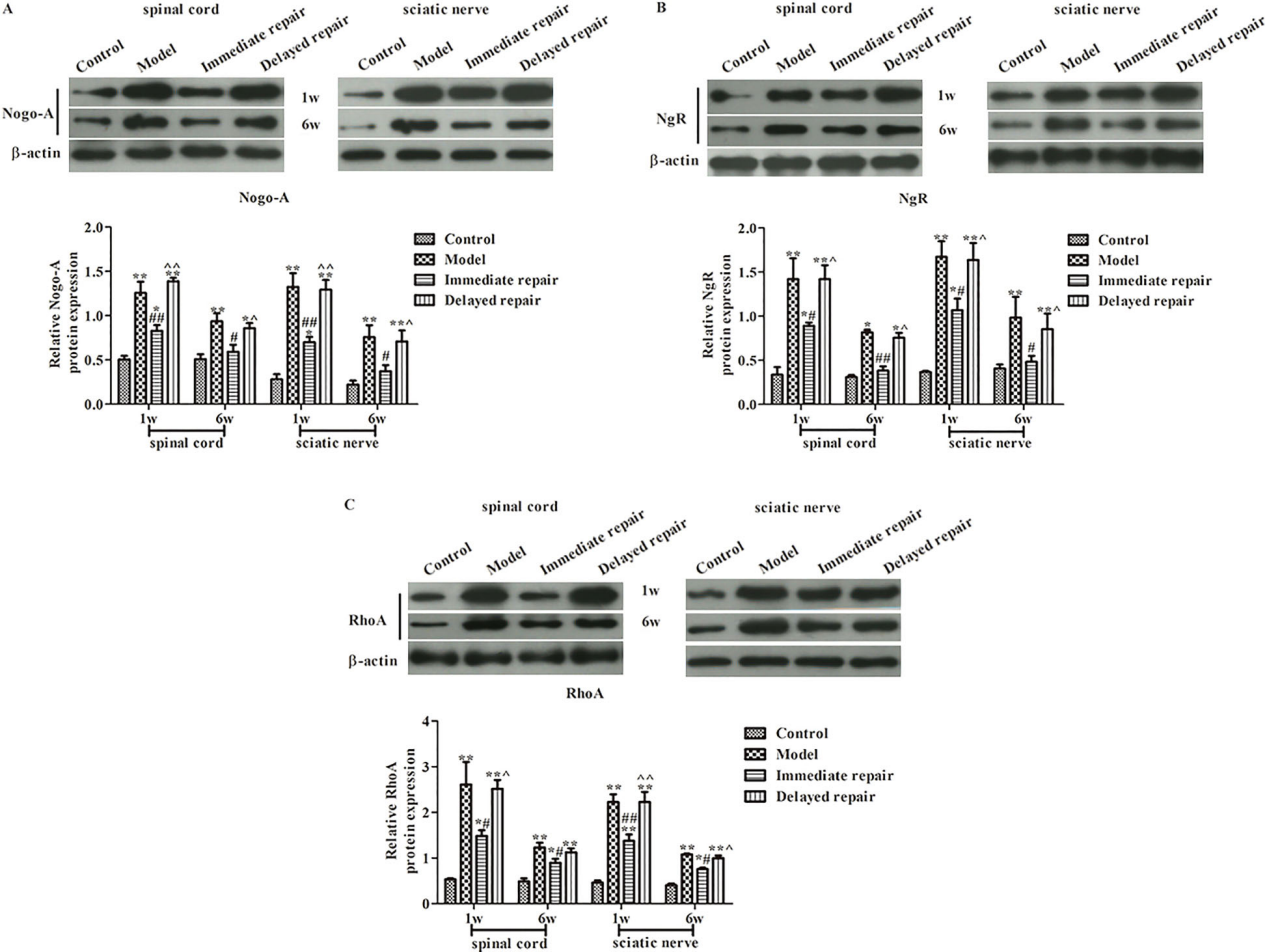
The expression of Nogo-A (**A**), NgR (**B**), and RhoA (**C**) protein was detected by western blot in rats after sciatic nerve injury. *P<0.05, **P<0.01 compared with control group; ^#^P<0.05, ^##^P<0.01 compared with model group; ^P<0.05, ^^P<0.01 compared with Immediate repair group. The data are reported as means±SD and were compared with ANOVA followed by LSD test.

As shown in Figure 4, 1 week after injury, compared with the control group, the protein expressions of Nogo-A (Figure 4A), NgR (Figure 4B), and RhoA (Figure 4C) in spinal cord and sciatic nerve tissues of the other three groups were significantly increased (P<0.05). Compared with the model group, the expressions of Nogo-A, NgR, and RhoA proteins in the immediate repair group was significantly decreased (P<0.05). After 6 weeks from injury, compared with the control group, the protein expressions of Nogo-A, NgR, and RhoA in the other three groups were higher (P<0.05). Compared with the model group, the protein expressions of Nogo-A, NgR, and RhoA in the immediate repair group and the delayed repair group were significantly decreased, and the protein expressions of Nogo-A, NgR, and RhoA in the immediate repair group were lower than those in the delayed repair group (P<0.05).

## Discussion

Nerve regeneration is a complex biological process, including a variety of cells, growth factors, and extracellular matrix. Elucidating the molecular mechanism of peripheral nerve injury and nerve repair can greatly improve the therapeutic strategies of the disease (16,17). Therefore, the search for the key genes that activate the intrinsic growth ability of neurons is important for the field. Peripheral neuropathy of the lower extremity is a common condition, usually associated with trauma and surgery (18,19). These injuries seriously affect the activity and function of patients, and are closely related to social and economic problems.

Previous studies have demonstrated the expression of Nogo-A in peripheral sensory neurons, the increase of Nogo-A mRNA or protein in hippocampal pyramidal neurons after injury of the inner olfactory cortex, and the increase of Nogo-A mRNA or protein in neurons after middle cerebral artery occlusion (20–22). In our study, the expression of Nogo-A protein in the spinal cord and sciatic nerve tissues increased after sciatic nerve transection. Compared with the sciatic nerve transection group, the pathological changes in the immediately repaired sciatic nerve group were less, and the protein expressions of Nogo-A, NgR, and RhoA in the spinal cord and sciatic nerve tissues were decreased. RhoA is described in experimental models of sciatic nerve injury and central nervous system myelin exposure, indicating that the pharmacological blockers of RhoA block the function of Nogo-A (23,24). Our study suggests that RhoA signal was a key regulator of Nogo-A in the repair of sciatic nerve injury. Inhibition of RhoA or Rho kinase downstream from C3 transferase promotes axonal growth and axonal regeneration after injury, which is related to a decrease of Nogo-A transcription level (25,26). The regulation of Nogo-A expression by RhoA is closely related to axonal dynamics. Nogo-A restrains neuronal growth by negatively regulating the movement of the growth cone (27). Our study suggests that Nogo-A played a role in the inhibition of axon growth.

NgR is a common receptor for the three known myelin-associated inhibitors, Nogo-A, myelin-associated glycoprotein (MAG), and oligodendrocyte myelin glycoprotein (OMgp) (28). NgR plays a key role in the failure of axonal regeneration in the adult mammalian CNS (29). In previous studies, NgR antagonistic peptide NEP1-40, a soluble NgR, and a transgenic NgR inhibitor with soluble function blocked NgR fragment NGR310 (30). NgR has been used to antagonize the binding of myelin-related inhibitors with NgR, thus promoting axon regeneration and functional recovery after spinal cord injury (31,32). The inhibitory effect of NgR on the growth of myelin sheath has also been demonstrated *in vitro* (33). In this study, the protein expression of NgR in spinal cord and sciatic nerve tissues decreased compared with the sciatic nerve transection group. These findings suggest that Nogo-A and its receptor had significant effects on the repair of sciatic nerve injury in rats.

However, our study had some limitations. A lack of research on relevant pathways evaluated in our experiments prevented the elucidation of specific mechanisms by which Nogo-A inhibited the repair of sciatic nerve injury in rats.

In conclusion, the expression of Nogo-A and its receptor increased after sciatic nerve injury, suggesting that Nogo-A and its receptor may play an inhibitory role in the repair process of sciatic nerve injury in rats. Future studies may further evaluate the expression of Nogo A at the gene level and study its effect on the repair of sciatic nerve injury.

## References

[1] Bonin RP (2015). Running from pain: mechanisms of exercise-mediated prevention of neuropathic pain. Pain.

[2] Pan B, Huo T, Hu Y, Cao M, Bu X, Li Z (2020). Exendin-4 promotes Schwann cell proliferation and migration via activating the Jak-STAT pathway after peripheral nerve injury. Neuroscience.

[3] Chen YW, Li YT, Chen YC, Li ZY, Hung CH (2012). Exercise training attenuates neuropathic pain and cytokine expression after chronic constriction injury of rat sciatic nerve. Anesth Analg.

[4] Akyuz G, Kenis O (2014). Physical therapy modalities and rehabilitation techniques in the management of neuropathic pain. Am J Phys Med Rehabil.

[5] Dworkin RH, O'Connor AB, Backonja M, Farrar JT, Finnerup NB, Jensen TS (2007). Pharmacologic management of neuropathic pain: evidence-based recommendations. Pain.

[6] Almeida C, Maman DA, Kusuda R, Cadetti F, Ravanelli MI, Queiroz AL (2015). Exercise therapy normalizes BDNF upregulation and glial hyperactivity in a mouse model of neuropathic pain. Pain.

[7] Huber AB, Weinmann O, Brosamle C, Oertle T, Schwab ME (2002). Patterns of Nogo mRNA and protein expression in the developing and adult rat and after CNS lesions. J Neurosci.

[8] Mingorance-Le Meur A, Zheng B, Soriano E, del Rio JA (2007). Involvement of the myelin-associated inhibitor Nogo-A in early cortical development and neuronal maturation. Cereb Cortex.

[9] Hunt D, Coffin RS, Prinjha RK, Campbell G, Anderson PN (2003). Nogo-A expression in the intact and injured nervous system. Mol Cell Neurosci.

[B10] Cheatwood JL, Emerick AJ, Schwab ME, Kartje GL (2008). Nogo-A expression after focal ischemic stroke in the adult rat. Stroke.

[11] Mingorance A, Fontana X, Solé M, Burgaya F, Urena JM, Teng FY (2004). Regulation of Nogo and Nogo receptor during the development of the entorhino-hippocampal pathway and after adult hippocampal lesions. Mol Cell Neurosci.

[12] Vajda F, Jordi N, Dalkara D, Joly S, Christ F, Tews B (2015). Cell type-specific Nogo-A gene ablation promotes axonal regeneration in the injured adult optic nerve. Cell Death Differ.

[13] Petrinovic MM, Duncan CS, Bourikas D, Weinman O, Montani L, Schroeter A (2010). Neuronal Nogo-A regulates neurite fasciculation, branching and extension in the developing nervous system. Development.

[14] Petrinovic MM, Hourez R, Aloy EM, Dewarrat G, Gall D, Weinmann O (2013). Neuronal Nogo-A negatively regulates dendritic morphology and synaptic transmission in the cerebellum. Proc Natl Acad Sci USA.

[15] Papadopoulos CM, Tsai SY, Cheatwood JL, Bollnow MR, Kolb BE, Schwab ME (2006). Dendritic plasticity in the adult rat following middle cerebral artery occlusion and Nogo-a neutralization. Cereb Cortex.

[16] Cobianchi S, Marinelli S, Florenzano F, Pavone F, Luvisetto S (2010). Short- but not long-lasting treadmill running reduces allodynia and improves functional recovery after peripheral nerve injury. Neuroscience.

[17] Groover AL, Ryals JM, Guilford BL, Wilson NM, Christianson JA, Wright DE (2013). Exercise-mediated improvements in painful neuropathy associated with prediabetes in mice. Pain.

[18] Ma CHE, Takao O, Cobos EJ, Latrémoliàre A, Ghasemlou N, Brenner GJ (2011). Accelerating axonal growth promotes motor recovery after peripheral nerve injury in mice. J Clin Investig.

[19] Li L, Li Y, Fan Z, Wang X, Li Z, Wen J (2019). Ascorbic acid facilitates neural regeneration after sciatic nerve crush injury. Front Cell Neurosci.

[20] Pradhan AD, Case AM, Farrer RG, Tsai SY, Cheatwood JL, Martin JL (2010). Dendritic spine alterations in neocortical pyramidal neurons following postnatal neuronal Nogo-A knockdown. Dev Neurosci.

[B21] Delekate A, Zagrebelsky M, Kramer S, Schwab ME, Korte M (2011). NogoA restricts synaptic plasticity in the adult hippocampus on a fast time scale. Proc Natl Acad Sci USA.

[22] Aloy EM, Weinmann O, Pot C, Kasper H, Dodd DA, Rulicke T (2006). Synaptic destabilization by neuronal Nogo-a. Brain Cell Biol.

[23] Liu YY, Jin WL, Liu HL, Ju G (2003). Electron microscopic localization of Nogo-A at the postsynaptic active zone of the rat. Neurosci Lett.

[24] Schwab ME (2010). Functions of Nogo proteins and their receptors in the nervous system. Nat Rev Neurosci.

[25] Fournier AE, GrandPre T, Strittmatter SM (2001). Identification of a receptor mediating Nogo-66 inhibition of axonal regeneration. Nature.

[26] Wang KC, Kim JA, Sivasankaran R, Segal R, He Z (2002). P75 interacts with the Nogo receptor as a co-receptor for Nogo, MAG and OMgp. Nature.

[27] Mi S, Lee X, Shao Z, Thill G, Ji B, Relton J (2004). LINGO-1 is a component of the Nogo-66 receptor/p75 signaling complex. Nat Neurosci.

[28] Shao Z, Browning JL, Lee X, Scott ML, Shulga-Morskaya S, Allaire N (2005). MiTAJ/TROY, an orphan TNF receptor family member, binds Nogo-66 receptor 1 and regulates axonal regeneration. Neuron.

[29] Brann AB, Tcherpakov M, Williams IM, Futerman AH, Fainzilber M (2002). Nerve growth factor-induced p75-mediated death of cultured hippocampal neurons is age-dependent and transduced through ceramide generated by neutral sphingomyelinase. J Biol Chem.

[30] Karnezis T, Mandemakers W, McQualter JL, Zheng B, Ho PP, Jordan KA (2004). The neurite outgrowth inhibitor Nogo A is involved in autoimmune-mediated demyelination. Nat Neurosci.

[31] Bros-Facer V, Krull D, Taylor A, Dick JRT, Bates SA, Cleveland MS (2014). Treatment with an antibody directed against Nogo-A delays disease progression in the SOD1G93A mouse model of Amyotrophic lateral sclerosis. Hum Mol Genet.

[32] Oertle T, van der Haar ME, Bandtlow CE, Robeva A, Burfeind P, Buss A (2003). Nogo-A inhibits neurite outgrowth and cell spreading with three discrete regions. J Neurosci.

[33] Joset A, Dodd DA, Halegoua S, Schwab ME (2010). Pincher-generated Nogo-A endosomes mediate growth cone collapse and retrograde signaling. J Cell Biol.

